# Investigation of the relationship between blood lactate levels and neonatal hyperbilirubinemia: A predictive approach to assessing severity on NHB in neonates

**DOI:** 10.5937/jomb0-54717

**Published:** 2025-11-05

**Authors:** Kun Shao, Yun-Heng Zhou, Gang-Qiang Zhang

**Affiliations:** 1 The First Affiliated Hospital of Fuyang Normal University, Fuyang, Anhui, 236000, China; 2 Clinical Laboratory, Fuyang Women and Children's Hospital, Fuyang, Anhui, 236000, China; 3 Medical College of Fuyang Normal University, Fuyang, Anhui, 236000, China

**Keywords:** neonatal hyperbilirubinemia, blood lactic acid, total bilirubin, aspartate aminotransferase, creatine kinase isoenzyme, p2-microglobulin, dehydrogenation of glucose-6-phosphate, neonatalna hiperbilirubinemija, mlečna kiselina u krvi, ukupni bilirubin, aspartat aminotransferaza, kreatin-kinaza izoenzim, p2-mikroglobulin, glukoza-6-fosfat dehidrogenaza

## Abstract

**Background:**

Neonatal hyperbilirubinemia (NHB) severity is traditionally assessed using serum total bilirubin levels alone; however, bilirubin measurement can be influenced by multiple physiological factors, limiting its accuracy. This study aimed to explore the correlation between blood lactate levels and NHB and evaluate whether blood lactate could serve as a novel, objective biomarker for predicting the severity and associated organ dysfunction in neonates with NHB.

**Methods:**

A total of 123 children diagnosed with NHB and admitted to the obstetrics department from October 2021 to October 2022 were selected as the NHB group, and according to the severity of the disease, they were divided into mild, moderate, and severe. Fifty healthy neonates born in the department of obstetrics were selected as the control group. The levels of glucose-6-phosphate dehydrogenase (G6PD), creatine kinase isoenzyme (CK-MB), p2-microglobulin (P2-MG), aspartate aminotransferase (AST), blood lactic acid and total bilirubin in serum were compared. Pearson correlation analysis was used to analyse the correlation between blood lactate level and bilirubin level, and the ROC curve was used to determine the predictive value of blood lactate level for the severity of NHB in neonates.

**Results:**

The NHB group had significantly higher levels of AST, CK-MB, p2-MG, blood lactic acid, and total bilirubin than the NHB group (P&lt; 0.05). The G6PD level was significantly lower (P &lt; 0 .0 5 ). Pearson correlation analysis showed a positive correlation between blood lactate and total bilirubin levels (r= 0.604, P&lt; 0.001). ROC curve analysis indicated that blood lactate had superior predictive accuracy (AU C= 0.873, sensitivity = 81.3% , specificity = 86.0%) for assessing NHB severity compared to total bilirubin alone (AU C= 0.759, sensitivity = 86.2% , specificity = 82.0% ; P&lt;0.05).

**Conclusions:**

Neonates with NHB have higher serum levels of AST, CK-MB, p2-MG, blood lactate, and total bilirubin, while lower G6PD levels. The serum level of blood lactate is positively correlated with the total bilirubin level, which can be used to observe the severity of NHB in neonates.

## Abstract (sr)

**Uvod:**

Ozbiljnost neonatalne hiperbilirubinemije (NHB) se tradicionalno procenjuje isključivo na osnovu nivoa ukupnog bilirubina u serumu; međutim, merenje bilirubina može biti pod uticajem brojnih fizioloskih faktora, sto ograničava preciznost. Ova studija imala je za cilj da ispita korelaciju između nivoa laktata u krvi i NHB, kao i da proceni da li laktat u krvi može poslužiti kao novi, objektivni biomarker za predviđanje težine oboljenja i pridruženih ostećenja organa kod novorođenčadi sa NHB.

**Metode:**

Kao NHB grupa su izabrana ukupno 123 deteta sa dijagnostikovanom NHB koja su primljena u odeljenje akuserstva u periodu od oktobra 2021. do oktobra 2022. godine. Na osnovu težine bolesti podeljena su na blagu, umerenu i tesku formu. Pedeset zdravih novorođenčadi rođenih u akuserskom odeljenju činilo je kontrolnu grupu. Upoređivani su nivoi glukoza-6-fosfat dehidrogenaze (G6PD), kreatin-kinaze izoenzima (CK-MB), p2-mikroglo-bulina (P2-MG), aspartat aminotransferaze (AST), mlečne kiseline u krvi i ukupnog bilirubina u serumu. Pirsonova korelaciona analiza je korisćena za analizu povezanosti između nivoa laktata u krvi i nivoa bilirubina, dok je ROC kriva korisćena za utvrđivanje prediktivne vrednosti laktata u krvi za procenu težine NHB kod novorođenčadi.

**Rezultati:**

Grupa sa NHB imala je značajno vise nivoe AST, CK-MB, p2-MG, mlečne kiseline u krvi i ukupnog bilirubina u poređenju sa kontrolnom grupom (P&lt; 0,05). Nivo G6PD bio je značajno niži (P&lt; 0,05). Pirsonova analiza korelacije je pokazala pozitivnu povezanost između nivoa laktata u krvi i ukupnog bilirubina (r= 0,604, P&lt; 0,001). Analiza ROC krive pokazala je da laktat u krvi ima vecu prediktivnu tačnost (AU C= 0,873, osetljivost = 81,3% , specifičnost = 86,0%) za procenu težine NHB u poređenju sa samim ukupnim bilirubinom (AU C= 0,759, osetljivost = 86,2%, specifičnost = 82,0% ; P&lt; 0,05).

**Zaključak:**

Novorođenčad sa NHB imaju vise serumske nivoe AST, CK-MB, p2-MG, mlečne kiseline u krvi i ukupnog bilirubina, dok imaju niže nivoe G6PD. Serumski nivo mlečne kiseline u krvi je u pozitivnoj korelaciji sa nivoom ukupnog bilirubina i može se koristiti za procenu težine NHB kod novorođenčadi.

## Introduction

Neonatal hyperbilirubinemia mainly occurs in the newborn period, and its clinical symptoms are higher than the total serum bilirubin levels. Due to the young age of newborns after birth, the blood-brain development barrier in the brain is incomplete, and bilirubin, without binding serum proteins, can penetrate the blood-brain barrier into the basal ganglia. This results in irreversible nervous system damage in children, which seriously threatens their lives and health [1]. Studies have found [2] that about 50% of full-term newborns will develop jaundice, and 80% of premature infants and 60% of full-term newborns will develop NHB within seven days after birth. Without timely and effective treatment, persistent NHB can damage the body organs and physiological structure of the nervous system. Currently, the severity of NHB is mainly determined by neonates' serum total bilirubin levels in clinical practice. However, due to the influence of organ damage in children, the serum total bilirubin level is quite different from the actual condition of NHB, which can easily cause missed diagnosis and misdiagnosis in clinical practice. Therefore, it is urgent to find stable and objective diagnosis and treatment evidence [3].

Recent studies have begun exploring alternative biomarkers to improve NHB assessment, mainly focusing on metabolic indicators. Blood lactate, a key intermediate in glucose metabolism, has been identified as a sensitive marker of tissue hypoxia and organ dysfunction [4]. Several recent studies have highlighted the association between elevated lactate levels and neonatal conditions involving metabolic stress or organ dysfunction. For instance, the study reported a significant relationship between elevated blood lactate levels and neonatal hyperbilirubinemia severity, suggesting lactate as a potential early biomarker (PMID: 38765909). Another recent international study indicated that increased lactate levels correlate strongly with bilirubin-induced mitochondrial dysfunction, emphasising lactate's potential predictive value for organ injury in NHB (PMID: 26824991). Despite these findings, domestic research remains limited regarding lactate's predictive capability for NHB severity.

Compared to traditional biomarkers, blood lactate provides several advantages: it is less influenced by extraneous factors such as hemolysis and bilirubin metabolism complexities, reflects real-time tissue hypoxia, and thus objectively indicates organ function impairment at early disease stages. Hence, blood lactate may offer clinicians a more reliable and early indication of NHB severity, potentially facilitating timely intervention and improved neonatal outcomes. This study aims to address existing research gaps by comprehensively investigating the correlation between blood lactate and total bilirubin in neonates with NHB and evaluating the clinical utility of blood lactate levels in predicting NHB severity, ultimately supporting more accurate early clinical decision-making.

## Materials and methods

### Information

A total of 123 neonates diagnosed with neonatal hyperbilirubinemia from October 2021 to October 2022 were selected as the NHB group, including 62 males and 61 females. The mean gestational age was 39.12±1.21 weeks (37-42 weeks). The mean age was 4.00±2.37 days (1-15 days). The mean birth weight was (3.41 ±0.25) kg (2.54-3.71 kg). According to the degree of disease, the children were divided into mild (221.00-256.49 μmol/L), moderate (256.50-342.00 μmol/L), and severe (>342.00 μmol/L). During the same period, 50 healthy neonates born in the obstetrics department were selected as the control group. There were no differences in information such as gender and gestational age of the subjects (P>0.05), Table 1.

**Table 1:** Information on subjects. Inclusion criteria: ® consistent with the diagnosis of NHB in »Expert consensus on the diagnosis and treatment of neonatal hyperbilirubinemia« [5]; © term neonates; © jaundice within 24 hours after birth; @ recurrent jaundice after disappearance; © serum bilirubin > 221.00 μmol/L or increased more than 85.00 μmol/L daily.<br>Exclusion criteria: ® congenital malformation; © intrauterine distress and infection; © pathological jaundice caused by drugs and infection; @ severe organ failure; © patients with incomplete clinical data.

Group of groups	n	Gender	Gestational age	Age at birth	Birth weight
		(Male/Female)	(x̄±s, weeks)	(d, x̄±s)	(kg, x̄±s)
Control group	50	26/24	39.08±0.78	3.50±1.39	3.31±0.17
Mild group	42	22/20	38.66±0.72	3.92±1.55	3.27±0.15
Moderate group	41	20/21	38.79±0.72	4.15±1.44	3.25±0.13
Severe group	40	20/20	39.02±0.73	3.66±1.64	3.35±0.14
*t*		0.285	0.569	0.631	0.994
*P*		0.937	0.846	0.924	0.263

### Research methods

Early morning fasting venous blood (5 mL) was collected from each subject using standard sterile techniques. Samples were centrifuged at 1000 X g for 10 minutes to separate the serum. The serum levels of AST, CK-MB, P2-MG, and G6PD were measured via immunoturbidimetry using an automatic biochemical analyser (Hitachi 7080, Beckman Coulter, USA). Reagents and assay kits were provided by Suzhou Jima Biological Co., Ltd. (lot numbers have been documented and are available upon request to ensure reproducibility). Additionally, 0.5 mL of arterial blood was drawn under sterile conditions for the analysis of blood lactate and total bilirubin. These parameters were assessed using a GEM4000 automatic blood gas, electrolyte, and biochemical analyser (Instrumentation Laboratory, USA), following the manufacturer's instructions. All blood samples were processed within 30 minutes of collection to ensure result accuracy. Trained technicians conducted all procedures in accordance with standard operating protocols.

### Observation indicators and test standards

(1) Grouping criteria; Mild group: total bilirubin level 221.00-256.49 μmol/L; Moderate group: total bilirubin level: 256.50-342.00 μmol/L; Severe group: total bilirubin level >342.00 μmol/L;

(2) The levels of AST, CK-MB, β2-MG, and G6PD were compared;

(3) Pearson correlation analysis was used to analyse the correlation between blood lactic acid level and total bilirubin level;

(4) The ROC curve was used to analyse the predictive value of blood lactic acid concentration for the severity of NHB. Then, the predictive value of the total bilirubin level for the seriousness of NHB was analysed.

### Statistical methods

Data were calculated using SPSS 26.0. Measurement data were expressed as mean ± standard deviation (x̄±s). Comparisons between two groups were performed by independent-sample t-test, and multiple-group comparisons were performed by one-way ANOVA followed by LSD post-hoc tests. Categorical data were expressed as [n (%)] and analysed using the Chi-square (χ^2^) test. Pearson correlation analysis assessed correlations. ROC curves evaluated predictive accuracy, and the optimal cut-off values were determined using the Youden Index. Statistical significance was defined as P<0.05.

## Results

### The results of the blood indexes of the four groups were compared

**Figure 1:**
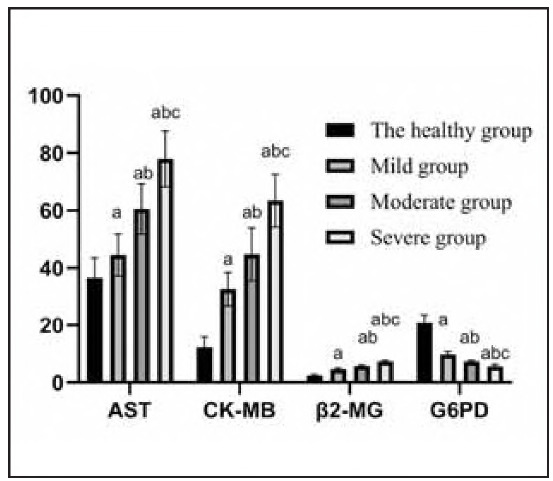
Bar graph of AST, CK-MB, β2-MG, and G6PD levels.

Serum AST, CK-MB, and β2-MG levels were higher, and G6PD levels were lower in the NHB group (P<0.05), Table 2, Figure 1.

**Table 2:** AST, CK-MB, β2-MG, G6PD levels (x̄±s). Note: a compared with the control group, b compared with the mild group, c compared with the moderate group, P<0.05

Group of groups	n	AST U/L)	CK-MB (U/L)	2-MG (mg/L)	G6PD (U/gHb)
Control group	50	36.54±6.93	12.31±3.57	2.43±0.40	20.80±2.82
Mild group	42	44.50±7.40^a^	32.62±5.80^a^	4.59±0.42^a^	9.78±0.97^a^
Moderate group	41	60.60±8.72^ab^	44.75±9.25^ab^	5.78±0.34^ab^	8.61±0.72^ab^
Severe group	40	77.96±9.75^abc^	63.47±9.15^abc^	7.34±0.35^abc^	5.65±0.61^abc^
*F*		48.936	59.453	8.256	11.874
*P*		0.001	0.001	0.001	0.001

### Blood lactic acid and serum total bilirubin levels

The levels of lactic acid and total bilirubin were higher in the NHB group (P<0.05), Table 3.

**Table 3:** AST, CK-MB, β2-MG, G6PD levels (x̄±s). Note: a compared with the control group, b compared with the mild group, c compared with the moderate group, P<0.05

Group of groups	n	LAC (mmol/L)	TBIL (μmol/L)
Control group	50	2.61±0.34	134.42±6.29
Mild group	42	3.11±0.32^a^	226.71±6.81^a^
Moderate group	41	3.89±0.53^ab^	302.49±15.98^ab^
Severe group	40	4.15±0.80^abc^	379.52±19.86^abc^
*F*		5.264	675.468
*P*		<0.001	<0.001

### Correlation between blood lactate level and total bilirubin level

**Figure 2:**
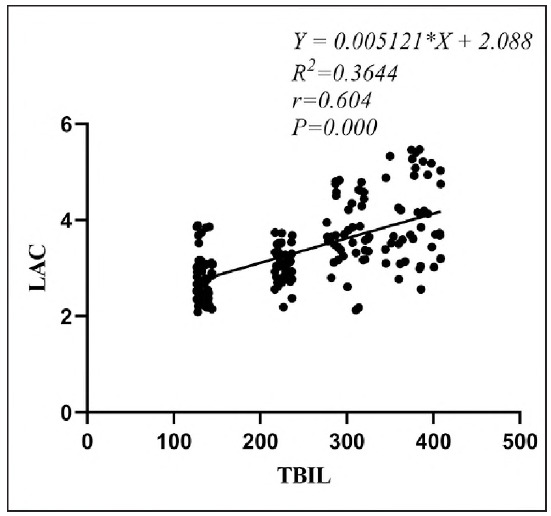
Correlation between blood lactate and total bilirubin.

Pearson bivariate linear correlation test showed that blood lactic acid level was positively correlated with total bilirubin level (r=0.604, P<0.05), as shown in Figure 2.

### To observe the predictive value of blood lactic acid and total bilirubin for NHB

**Figure 3:**
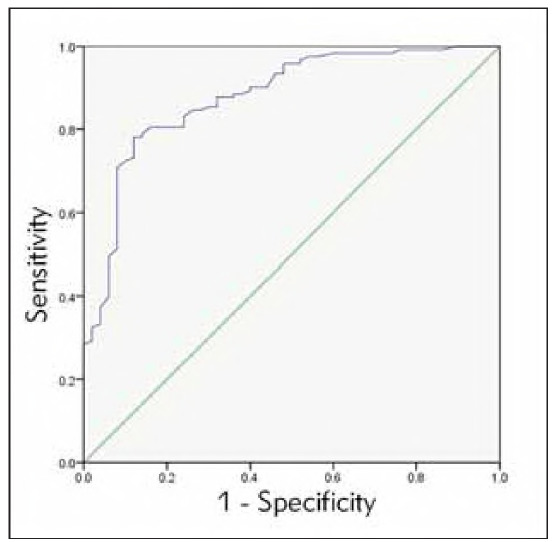
ROC curve of blood lactate.

**Figure 4:**
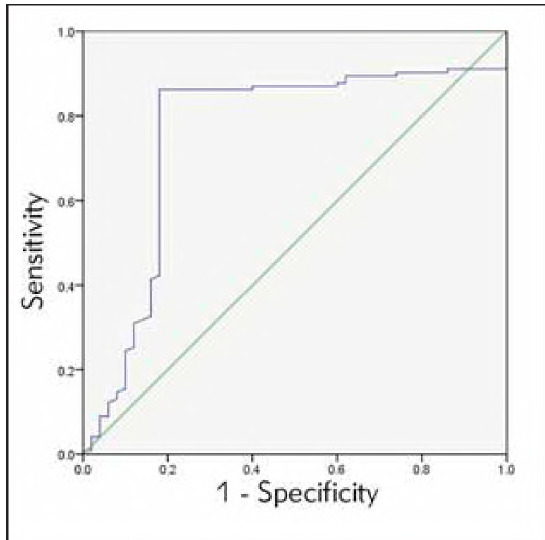
ROC curve of total bilirubin.

ROC showed that the blood lactic acid level of 2.93 mmol/L and a total bilirubin level of 201.67 μmol/L was used as the critical value, and NHB newborns were used as the state variable. The predictive value of the two indicators for NHB was observed, and the ROC curve was drawn, Table 4-Table 5, Figure 3-Figure 4.

**Table 4:** Predictive value of blood lactating alone.

Indicators	AUC	sensitivity	specificity	P	95%CI	
					Lower<br>limit	Upper<br>limit
LAC	0.873	81.30%	86.00%	0.000	0.805	0.940

**Table 5:** Predictive value of total bilirubin.

Indicators	AUC	sensitivity	specificity	*P*	95%CI	
					Lower<br>limit	Upper<br>limit
TBIL	0.759	86.20%	82.00%	0.000	0.669	0.848

## Discussion

NHB is among the most common diseases in paediatrics, and its incidence ranks first in neonatal diseases [6]. Unconjugated bilirubin, a heme metabolite from red blood cell breakdown, is converted in the liver to direct bilirubin and excreted via the duodenum, primarily through faeces [7]. Under normal conditions, bilirubin levels are low and exhibit antioxidant properties. However, elevated bilirubin can cross the blood-brain barrier, leading to neurotoxicity and damage to cardiac, hepatic, and gastrointestinal functions [8]. Studies have shown that excessive bilirubin may impair organ metabolism and mitochondrial function, resulting in lactic acid accumulation, hyperlactatemia, and an increased risk of kernicterus [9]. Clinically, monitoring total bilirubin levels in neonates is essential for early detection and prevention of NHB. This study observed significantly elevated AST, CK-MB, β2-MG, and decreased G6PD levels in the NHB group (P<0.05), highlighting their potential value in early identification and intervention.

Although serum total bilirubin is commonly used to assess NHB severity, its accuracy is limited due to various external factors. Recent studies suggest that blood lactate, a marker of metabolic stress and tissue hypoxia, may serve as a more reliable biomarker in NHB [4] [5]. However, research on its clinical utility remains limited, especially in domestic contexts. Our findings revealed a significant positive correlation between blood lactate and total bilirubin (r=0.604, P<0.001). ROC analysis demonstrated that blood lactate had superior predictive accuracy for NHB severity (AUC = 0.873, sensitivity = 81.3%, specificity = 86.0%), outperforming total bilirubin alone As an objective indicator of tissue hypoxia and organ dysfunction, blood lactate offers practical advantages for early clinical decision-making, independent of bilirubin metabolism.

Currently, the clinical diagnosis of neonatal hyperbilirubinemia (NHB) primarily relies on serum total bilirubin levels; however, there is no standardised approach for assessing disease severity or associated organ damage. Total bilirubin levels are susceptible to external influences, which may lead to discrepancies between measured values and actual bilirubin concentrations, thereby affecting early clinical diagnosis [10]. AST, predominantly found in the heart, liver, and skeletal muscle, increases in serum following cardiac or hepatic injury, making it a valuable marker for assessing organ damage [11]. CK-MB is a myocardial enzyme that can reflect the degree of myocardial injury to a certain extent [12]. The kidneys, which play a key role in NHB, often compensate until advanced damage occurs. Traditional renal markers such as blood urea nitrogen (BUN) and serum creatinine (Scr) rise only in later stages, necessitating more sensitive early indicators [13]. β2-microglobulin (β2-MG) is a good indicator of early renal function injury, which is more sensitive than BUN and Scr [14].

Additionally, glucose-6-phosphate dehydrogenase (G6PD), an enzyme essential for red blood cell protection, is associated with increased hemolysis and bilirubin levels when deficient [15]. This study found that levels of AST, CK-MB, and β2-MG were significantly higher, while G6PD levels were substantially lower in the mild, moderate, and severe NHB groups compared to the healthy group (P 0.05). These biomarkers can thus serve as early indicators of multiorgan involvement and provide an indirect assessment of NHB severity.

Analysis of the mechanism of increasing neonatal total bilirubin level: (1) The life span of neonatal red blood cells is short, only 70-90 days. The haemoglobin of ageing red blood cells is decomposed into heme, iron, and globin, and heme is converted into total bilirubin, but this is not the leading cause of inducing neonatal NHB [16]. (2) Immature liver function plays a key role. Low expression of the Y protein haplotype and underdeveloped bilirubin-conjugating enzyme systems reduce hepatic bilirubin uptake and conjugation, resulting in bilirubin accumulation and jaundice. This condition typically normalises within 5-10 days after birth [17]. Blood lactic acid is mainly produced in red blood cells, the brain, and striated muscle tissue. When the body functions normally, the content in the body is small [18]. Elevated lactate levels indicate tissue hypoxia or glucose metabolism disorders. NHB can disrupt the blood-brain barrier and impair liver function, leading to metabolic dysfunction and elevated blood lactate [19].

In this study, Pearson correlation analysis revealed a positive association between blood lactate levels and NHB severity (AUC = 0.604), suggesting that higher lactate correlates with more severe disease. Potential mechanisms include: (1) Elevated bilirubin increases endogenous carbon monoxide, promoting carboxyhemoglobin formation, which lacks oxygen-carrying capacity, causing tissue hypoxia and shifting glucose metabolism from aerobic to anaerobic, thereby increasing lactate production [20]. (2) High bilirubin impairs hepatocyte enzyme activity, particularly lactate dehydrogenase, reducing lactate clearance. Additionally, bilirubin-induced oxidative stress damages hepatocyte membranes, further impairing lactate metabolism [21]. (3) Excessive bilirubin may accumulate on mitochondrial membranes, triggering oxidative phosphorylation dysfunction and acute energy metabolism disturbances, leading to lactic acid buildup [22]. Therefore, blood lactate levels can be used to evaluate the severity of neonatal NHB to a certain extent.

ROC curve analysis in this study showed that the AUC of blood lactic acid level in predicting the severity of NHB was more significant than that of total bilirubin level, and the predictive value and accuracy were higher (P<0.05). Analysis of the causes and mechanisms: It may be because the metabolic process of total bilirubin is more complex, mainly from haemoglobin released after the destruction of red blood cells, but it also involves many factors such as transportation, uptake, excretion, and so on. Moreover, the causes of NHB show diversity [23], so it is not wise to use total bilirubin levels to predict the severity of NHB in children. Blood lactate level has a higher predictive value for the severity of NHB.

In conclusion, the levels of AST, CK-MB, β2-MG, and G6PD can serve as indicators of organ damage in neonates with NHB, while blood lactate levels show a significant correlation with serum total bilirubin and may be useful in predicting the severity of NHB in its early stages. However, this study has certain limitations, including a relatively small sample size and the inclusion of only full-term neonates. The exclusion of preterm infants limits the generalizability of the findings. Future studies with larger, more diverse neonatal populations are needed to validate these results and further explore the clinical utility of blood lactate as a predictive marker.

## Dodatak

### Acknowledgment

Not applicable.

### Funding

This work was supported by the Natural Science Research Project of Fuyang Normal University ((2020YXZX03ZD)). Study on the correlation between NSE, lactic acid level and neonatal hyperbilirubinemia.

### Ethics statement

This paper has been reviewed by relevant departments of our hospital, such as the Science and Education Department, Medical Department and Ethics Committee of Fuyang Women and Children's Hospital. The research content involved in this research meets the requirements of medical ethics and academic morality of our hospital, and the research content is reasonable, the risks are controllable, and there are no violations. The relevant research carried out is in line with the safe, standardised, and valid scientific research guiding principles and the clinical research ethics code requirements.

### Conflict of interest statement

All the authors declare that they have no conflict of interest in this work.
